# Apparent diffusion coefficient can assist in differentiating between benign and malignant primary bone tumors in pediatric patients

**DOI:** 10.1007/s00256-025-05060-8

**Published:** 2025-10-29

**Authors:** Yashas Ullas Lokesha, Shashi Bhushan Singh, Ricarda von Krüchten, Zahra Shokri Varniab, Manoj Kumar, Vidyani Suryadevara, Amir Hossein Sarrami, Tie Liang, Jason Wong, Allison Pribnow, Heike Elisabeth Daldrup-Link

**Affiliations:** 1 Department of Radiology, Stanford University School of Medicine, Stanford, CA 94305 USA; 2 Department of Pediatrics, Hematology/Oncology, Lucile Packard Children’s Hospital, Stanford University, Stanford, CA 94304 USA

**Keywords:** Apparent diffusion coefficient, Diffusion weighted imaging, Bone tumor, Pediatric, Benign, Malignant

## Abstract

**Objective:**

To evaluate differences in apparent diffusion coefficient (ADC) values between benign and malignant primary pediatric bone tumors and to assess their diagnostic accuracy in differentiating these tumors.

**Materials and methods:**

We retrospectively analyzed MRI scans of 96 pediatric patients (54 males, 42 females; mean age 12.97 ± 3.9 years) with primary bone tumors who underwent diffusion-weighted imaging, including 48 benign and 48 malignant tumors. We measured ADCmean, ADCmin, and ADCmax of the solid tumor part, carefully avoiding cystic, necrotic, or sclerosed tumor areas. The Wilcoxon rank-sum test was used to test the distributional difference of benign vs malignant tumors. ROC curve analysis was performed to assess the diagnostic accuracy. The optimal cutoff of ADC values to differentiate benign and malignant bone tumors was defined as the point at which the Youden index, the sum of sensitivity and specificity, was maximized.

**Results:**

The median values of the ADCmean, ADCmin, and ADCmax for benign bone tumors [1.34 (1.13–1.83), 0.98 (0.73–1.34), and 1.80 (1.57–2.46) × 10^−3^mm^2^/s, respectively] were significantly higher compared to malignant bone tumors [0.93 (0.78–1.03), 0.59 (0.43–0.72), and 1.35 (1.22–1.66) × 10^−3^mm^2^/s, respectively; all *p* < 0.05]. ADCmean yielded the highest diagnostic accuracy, with an optimal cutoff of 1.04 (0.94–1.15) × 10^−3^mm^2^/s (sensitivity 77%, specificity 93%, AUC = 0.91). An ADCmin cutoff of 0.82 (0.65–0.98) × 10^−3^mm^2^/s resulted in a sensitivity of 87.5%, specificity of 70.0%, and AUC of 0.85. An ADCmax cutoff of 1.48 (1.18–1.78) × 10^−3^mm^2^/s achieved a sensitivity of 68%, specificity of 81%, and AUC of 0.80.

**Conclusion:**

ADCmean, ADCmin, and ADCmax differ significantly between benign and malignant pediatric bone tumors, and the ADCmean provides the highest diagnostic accuracy.

**Supplementary Information:**

The online version contains supplementary material available at 10.1007/s00256-025-05060-8.

## Introduction

Malignant bone tumors constituted 12,213 reported cases among individuals aged 0–19 years in the United States between 2003 and 2019, with an overall age-adjusted incidence rate of 8.7 per million [1]. Distinguishing between benign and malignant pediatric primary bone tumors is crucial because benign tumors can often be observed, while malignant tumors must be referred to treatment without delay [2]. Plain radiographs have a sensitivity and specificity of 72.7% and 78.4%, respectively [3]. Conventional MRI has a sensitivity and specificity of 90.5% and 81.1%, respectively, to differentiate benign and malignant bone tumors [4].

Diffusion-weighted imaging (DWI) has been utilized to differentiate benign and malignant bone tumors in adult patients [5]. DWI visualizes the Brownian motion of water protons in bone tumors. The apparent diffusion coefficient (ADC) is a quantitative measure of Brownian motion and is a tumor cell density marker [6]. Several investigators reported that malignant bone tumors exhibit higher tumor cell density and lower ADC values than benign bone tumors in adult patients [5, 7, 8]. Ogawa et al. reported that benign bone tumors in adult patients exhibited significantly higher ADCmin values (1.5 ± 0.47 × 10^−3^ mm2/s) compared to malignant primary bone tumors and bone metastases (0.49 ± 0.17×10^−3^ mm^2^/s) [9]. In a study involving a mixed population of pediatric and adult patients, Rao et al. reported an ADCmean cutoff value of 1.31 × 10^−3^ mm^2^/s for differentiating benign bone lesions (including benign tumors and osteomyelitis) from malignant bone tumors [10]. Previous studies did not exclusively evaluate primary bone tumors in children.

Furthermore, the literature presents conflicting reports regarding whether ADCmean, ADCmin, or ADCmax yields superior results for tumor characterization. Neubauer et al. reported that ADCmean provided better differentiation between benign bone tumors and tumor-like lesions compared to malignant primary and metastatic bone tumors [11]. Ahlawat et al. reported that ADCmin better differentiated benign and malignant bone tumors in a mixed group of 20 adult and 11 pediatric patients [12]. Mansour et al. found in a cohort of 62 pediatric and adult patients that ADCmax was the most effective for distinguishing between benign and malignant bone tumors [13]. No prior study has systematically compared the sensitivity and specificity of ADCmean, ADCmin, and ADCmax in distinguishing between benign and malignant primary bone tumors in pediatric patients.

To close this gap, we evaluated differences in ADC values between benign and malignant primary pediatric bone tumors and assessed their diagnostic accuracy in differentiating these tumors.

## Materials and methods

### Study population

This retrospective study was approved by the Institutional Review Board (IRB). We analyzed MRI scans of pediatric patients at our institution using the following inclusion criteria: (1) MRI scan with DWI obtained between September 2012 and August 2024, (2) age ≤ 18 years, (3) MRI diagnosis of primary bone tumor (excluding bone cysts and osteochondroma), (4) newly diagnosed, untreated lesions, and (5) histopathology for malignant tumors or at least 12 months of follow-up for benign lesions.

Exclusion criteria included (1) any prior chemotherapy, radiation therapy, or surgical intervention, (2) the presence of pathologic fractures, and (3) image artifacts and/or incomplete imaging data preventing calculation of ADC values.

Applying these criteria yielded a final cohort of 96 patients, comprising 48 benign and 48 malignant tumors. Benign tumors included 28 fibrous dysplasias, 9 enchondromas, and 11 non-ossifying fibromas. Malignant tumors comprised 17 Ewing sarcomas, 9 chondroblastic osteosarcomas, and 22 osteoblastic osteosarcomas. All eligible cases meeting the inclusion and exclusion criteria were included in the analysis to minimize selection bias (Fig. S1).

### MR examination

All patients underwent an MRI on a 3-T Signa scanner (GE Healthcare, Milwaukee, WI) using appropriate surface coils. The imaging protocol included the following sequences: Non-fat-saturated T1-SE(spin echo), non-fat-saturated T2-FSE(fast spin echo), T2-FSE with fat saturation or short tau inversion recovery (STIR) sequence, Diffusion-weighted single-shot spin-echo echo-planar imaging (EPI) sequence with two *b*-values (*b* = 0–50 s/mm^2^ and *b* = 600–1000 mm^2^) and a postcontrast fat-saturated T1-weighted gradient-echo sequence after intravenous injection of gadobutrol (Gadavist, 0.1 mmol Gd/kg). Pulse sequence parameters for all sequences are listed in Table 1. ADC maps were automatically generated from the DWI sequences using the GE Signa scanner software. This process involved calculating the slope of the logarithmic signal intensity relative to the *b*-value, allowing quantitative assessment of the tissue diffusivity.

**Table 1:** MRI scan parameters for sequences used in the study. All values are presented as ranges observed across patients in the study cohort

Sequence	Slice thickness (mm)	Spacing (mm)	FOV (mm)	Matrix size (pixel)	TR (ms)	TE (ms)	Flip angle (°)
T1 Spin echo	3–6	3–6	140–360	168–512 × 168–370	545–995	5.34–17.7	90–160
T2 Fast spin echo	2–5	2–5.5	160–240	320–512 × 224–312	2960–6059	50.3–109	90–142
T2 Fat saturated Fast spin echo	3–6	3–6	140–420	156–416 × 192–415	2400–5323	76.3–88.2	90–180
Short tau inversion recovery	3–7	3–7	180–480	236–384 × 160–256	3800–6311	26.7–62	90–111
Diffusion weighted	3–7	3–7	160–380	80–128 × 80–128	2500–10,000	46.9–98.1	90
Post-contrast T1 gradient echo	0.7–3	0.5–2	140–480	168–416 × 192–416	4.1–9.5	1.7–5.2	12–90

### Image analysis

A general radiologist with 5 years of experience, blinded to the tumor diagnosis, clinical history, and histopathologic findings of the patients, reviewed all MRI examinations and delineated the bone lesion on DWI and ADC maps using an operator-defined region of interest (ROI). The ROI was carefully placed within the solid and homogeneous portion of the lesion, as identified on T2-weighted and contrast-enhanced images. Necrotic, cystic (T2-hyperintense, non-enhancing) and sclerosed (T1 and T2 hypointense) areas were excluded. The ADCmean, ADCmin, and ADCmax values were calculated with Sectra PACS (version 25.2.22.7826, © Sectra AB, Sweden).

### Statistical analysis

A Wilcoxon rank-sum test was used to compare the distribution of ADC values between benign and malignant bone tumors. For each tumor category, median ADC values along with interquartile ranges (IQR) were calculated and presented, minimizing the influence of outliers. Sensitivity and specificity were determined by comparing the classification results based on ADC cutoff values with the gold standard diagnosis. The gold standard was defined as histopathological findings for malignant lesions and either histopathological confirmation or imaging follow-up for at least 12 months for benign lesions. Sensitivity was calculated as the proportion of malignant cases correctly classified as malignant (true positives), and specificity was calculated as the proportion of benign cases correctly classified as benign (true negatives). The optimal ADC cutoff value was determined as the point at which the Youden index, the sum of sensitivity and specificity, was maximized to differentiate benign and malignant bone tumors. Receiver operating characteristic (ROC) curves were generated to evaluate the diagnostic accuracy of ADCmean, ADCmin, and ADCmax in differentiating benign from malignant bone tumors. The area under the curve (AUC) was calculated for each parameter, with higher AUC values indicating greater diagnostic accuracy. *p*-values of <0.05 were considered statistically significant. All statistical analyses were performed using the statistical package for the social sciences software (IBM SPSS Statistics for Mac, version 28.0, Armonk, NY: IBM Corp.). The plot was created using R version 4.4.1. specifically, the R package pROC version 1.18.5.

## Results

**Fig. 1:**
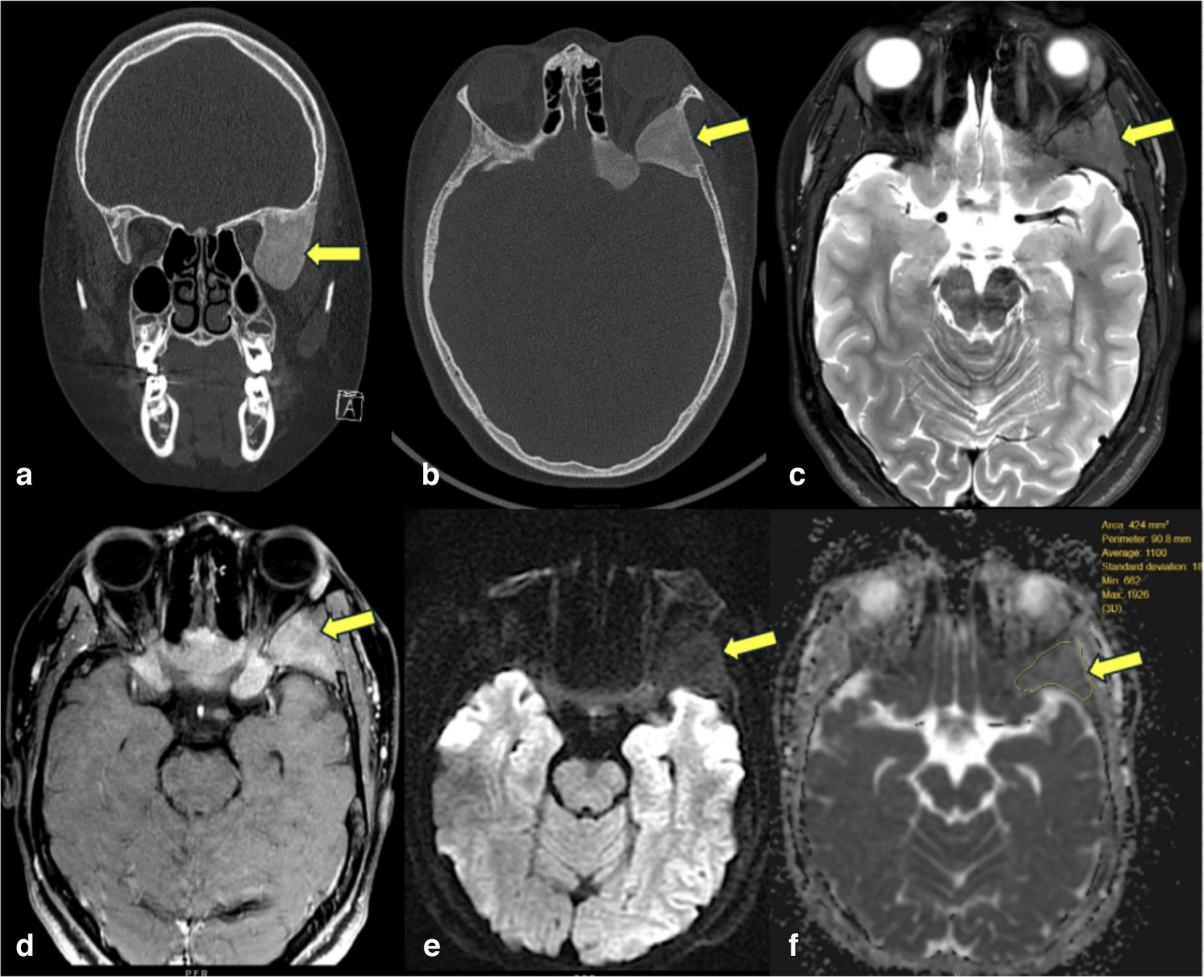
Fibrous dysplasia of the sphenoid bone in a 13-year-old male. **a** Coronal CT of the skull in bone window reveals an expansile lesion with ground glass attenuation (arrow) of the greater wing of the left sphenoid bone. **b** Axial CT image shows an expansile lesion with ground glass attenuation in the greater wing (arrow) and body of the left sphenoid. **c** Axial T2-weighted image demonstrates relatively low T2 signal of the lesion (arrow), consistent with fibrous stroma. **d** Axial post-contrast fat-suppressed T1-weighted image shows that the lesion enhances with contrast (arrow). **e** Axial DW-MRI reveals no restricted diffusion of the lesion (arrow). **f** Corresponding ADC map shows relatively low signal of the lesion, with ADCmean of 1.10 × 10^−3^ mm^2^/s ADCmin of 0.66 × 10^−3^ mm^2^/s, and ADCmax of 1.92 × 10^−3^ mm^2^/s

Fibrous dysplasia (Fig. 1) presented as moderately low signal intensity on T1-weighted imaging (T1WI), intermediate signal intensity on T2-weighted imaging (T2WI), and heterogeneous moderate enhancement on contrast-enhanced T1-weighted imaging (T1C+ [Gd]). Non-ossifying fibromas presented as low signal intensity on T1WI and intermediate to heterogeneously high signal intensity on T2WI, often with a low-signal peripheral rim. On T1C+ (Gd), lesions demonstrated heterogeneous enhancement. On ADC maps, seven fibrous dysplasia lesions and one non-ossifying fibroma lesion demonstrated lower signal intensity, while ten non-ossifying fibroma lesions and twenty-one fibrous dysplasia lesions demonstrated higher signal intensity than muscle, used as an internal standard.

**Fig. 2:**
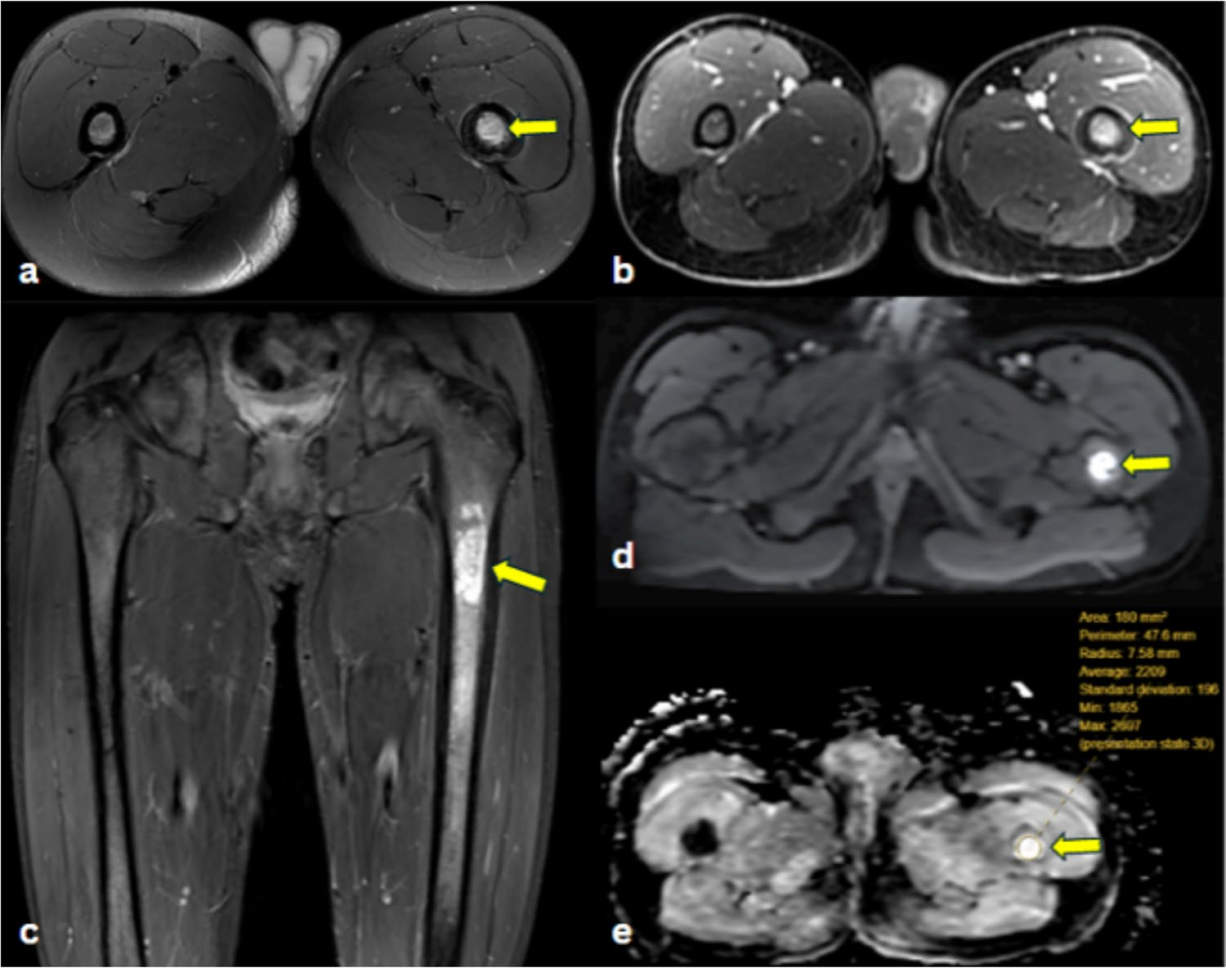
Enchondroma of left femur in a 15-year-old male. **a** Axial T2-fat-saturated image demonstrates an intramedullary hyperintense lesion in the mid-proximal left femur (arrow). **b** Axial post-contrast fat-suppressed T1-weighted image shows homogeneous contrast enhancement of the lesion (arrow). **c** Coronal short tau inversion recovery (STIR) MR image demonstrates a hyperintense subtrochanteric intramedullary multilobulated lesion in the mid-proximal left femur (arrow) with mild endosteal scalloping. **d** Axial DW-MRI reveals a hyperintense lesion (arrow) in the left proximal femur. **e** Corresponding ADC map shows hyperintense signal (arrow) of the lesion, indicating limited restricted diffusion, with ADCmean of 2.20 × 10^−3^ mm^2^/s, ADCmin of 1.86 × 10^−3^ mm^2^/s and ADCmax of 2.69 × 10^−3^ mm^2^/s

Enchondromas (Fig. 2) presented as intermediate-to-low signal intensity on T1WI, heterogeneously high signal intensity on T2WI, and moderate heterogeneous enhancement on T1C+ (Gd). On ADC maps, all lesions demonstrated higher signal intensity than muscle.

**Fig. 3:**
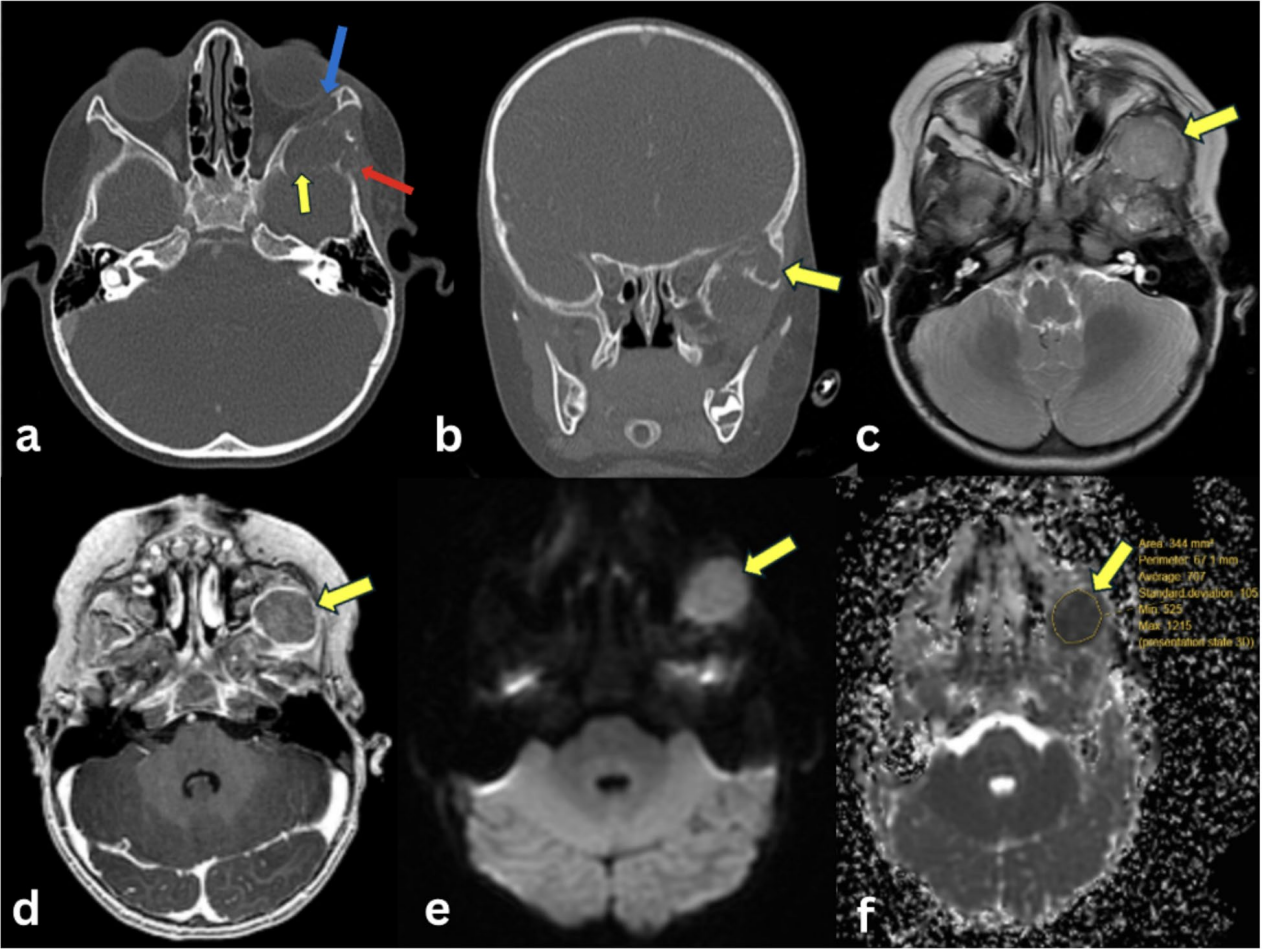
Ewing sarcoma of the sphenoid bone in a 17-month-old female. **a** Axial CT image of the skull in bone window demonstrates a lytic lesion involving the greater wing of the left sphenoid bone (yellow arrow) with erosion of the left lateral orbital wall (blue arrow) and squamous portion of the left temporal bone (red arrow). **b** Coronal reformatted CT image shows that the lytic lesion in the greater wing of the left sphenoid bone (arrow) extends into the left infratemporal fossa. **c** Axial T2-weighted image demonstrates a mild hyperintense signal of the lesion (arrow) in the left sphenoid bone. **d** Axial post-contrast fat-suppressed T1-weighted image demonstrates contrast-enhancement of the mass (arrow). **e** Axial DW-MRI reveals a hyperintense lesion (arrow), consistent with restricted diffusion. **f** Corresponding ADC map shows a hypointense signal (arrow), indicating restricted diffusion, with ADCmean of 0.70 × 10^−3^ mm^2^/s ADCmin of 0.52 × 10^−3^ mm^2^/s, and ADCmax of 1.21 × 10^−3^ mm^2^/s

Ewing sarcomas (Fig. 3) presented as low to intermediate signal intensity on T1WI, heterogeneously high signal intensity on T2WI, and moderate to high heterogeneous enhancement on T1C+ (Gd). On ADC maps, all lesions demonstrated lower signal intensity than muscle.

**Fig. 4:**
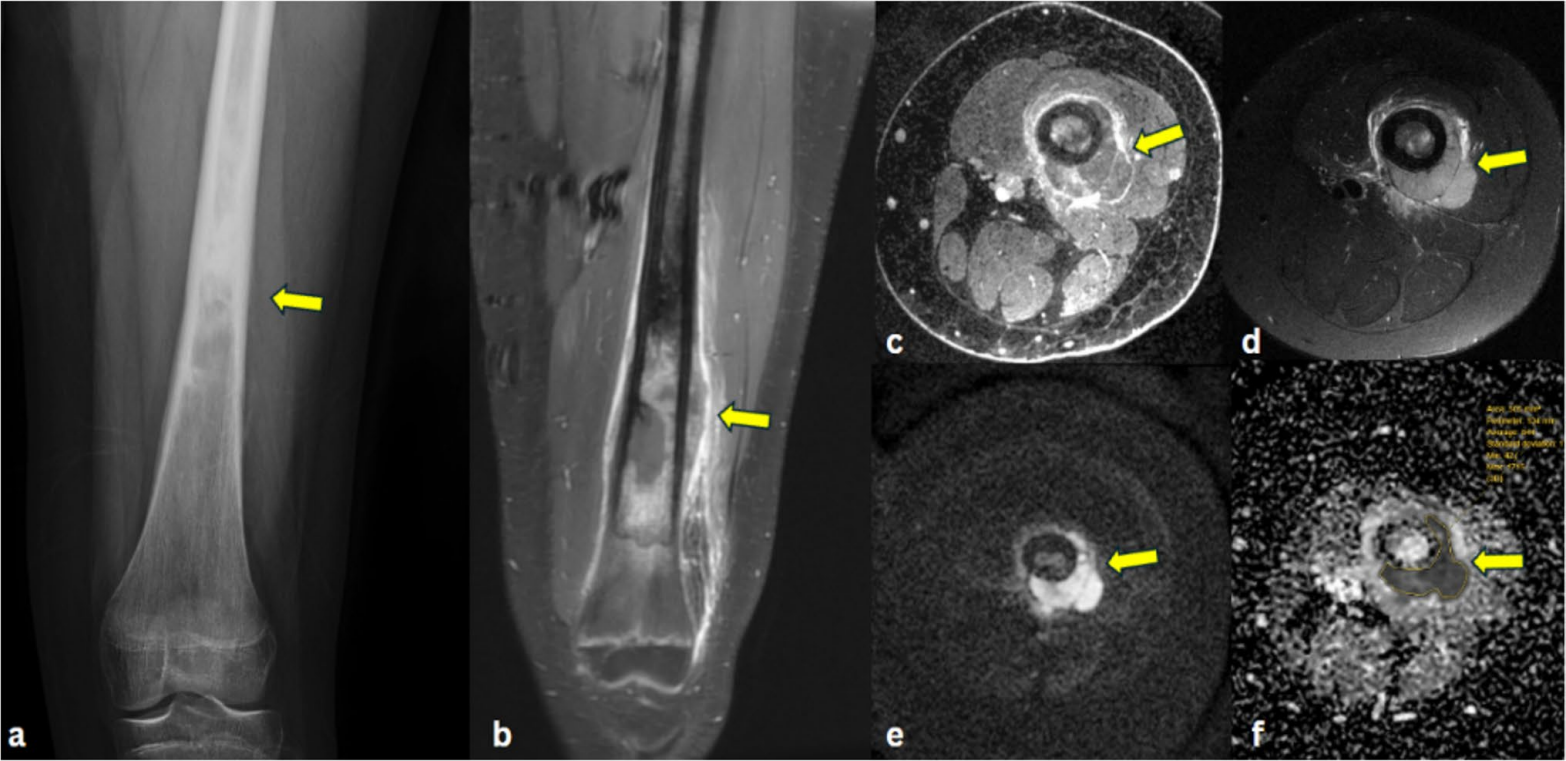
Osteoblastic osteosarcoma of the femur in a 14-year-old female. **a** AP radiograph of the left femur shows heterogeneous sclerosis (arrow) of the mid to distal femoral diaphysis with a wide zone of transition and aggressive periosteal reaction. **b** Coronal post-contrast fat-suppressed T1-weighted image shows that the lesion has an extraosseous soft tissue component (arrow). **c** Axial post-contrast fat-suppressed T1-weighted image shows inhomogeneous contrast enhancement of the intra- and extraosseous mass (arrow). **d** Axial T2-weighted fat-saturated image demonstrates relatively hypointense intramedullary tumor, consistent with sclerosis on the radiograph, and hyperintense extraosseous soft tissue mass (arrow). **e** Axial DW-MRI reveals intramedullary hypointense components and extraosseous hyperintense components of the lesion (arrow). **f** Corresponding ADC map shows hypointense signal of the extraosseous soft tissue mass (arrow), indicating restricted diffusion, with ADCmean of 0.84 × 10^−3^ mm^2^/s, ADCmin of 0.42 × 10^−3^ mm^2^/s, and ADCmax of 1.71 × 10^−3^ mm^2^/s

Osteoblastic osteosarcomas (Fig. 4) presented as inhomogeneous T1-hypointense (with non-mineralized components showing intermediate signal intensity and mineralized components showing low signal intensity), inhomogeneous T2-hyperintense (with non-mineralized components showing high signal intensity and mineralized components showing low signal intensity), and heterogeneous enhancement on T1C+ (Gd). On ADC maps, all lesions demonstrated lower signal intensity than muscle.

**Fig. 5:**
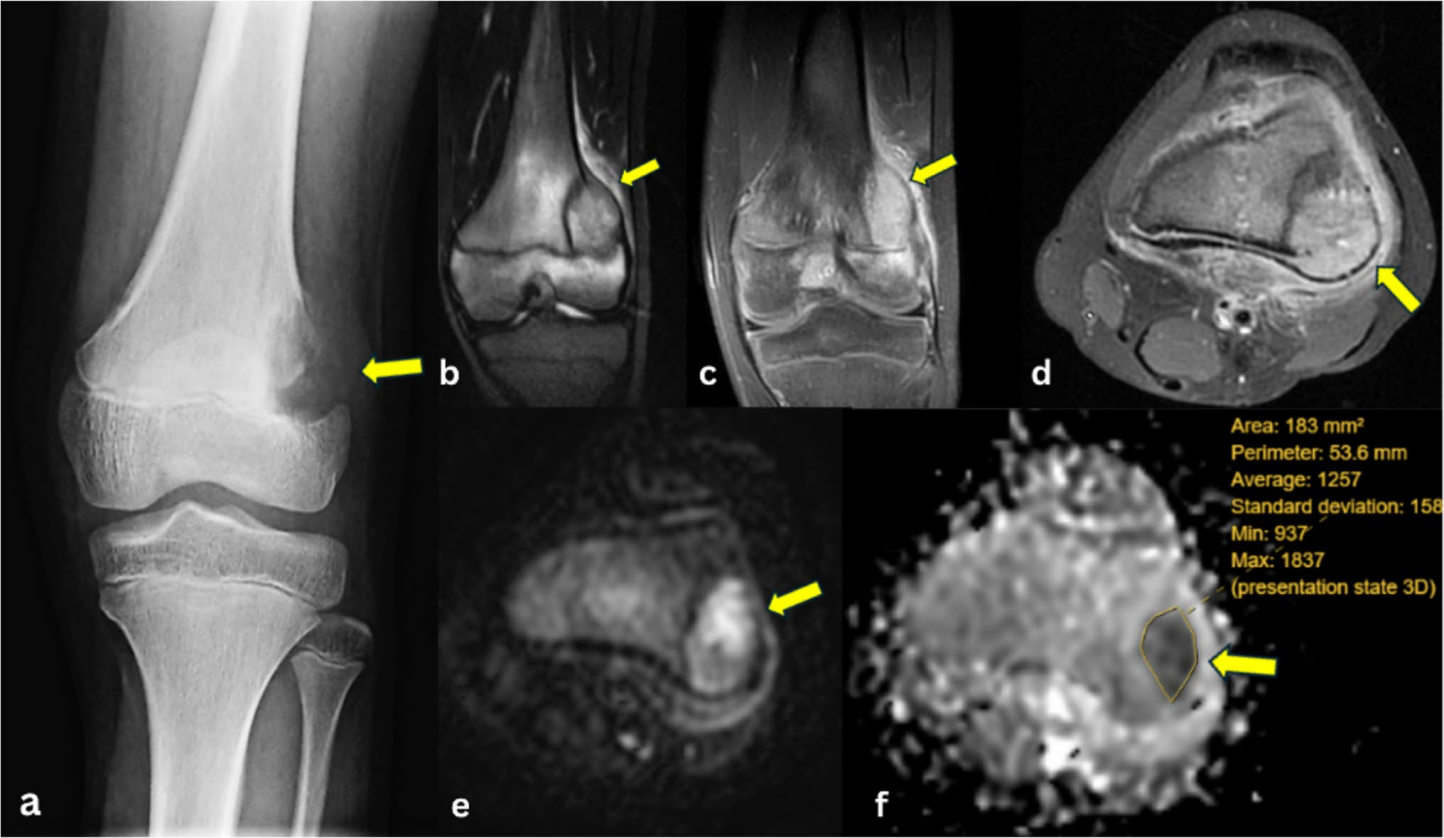
Chondroblastic osteosarcoma of the femur in a 13-year-old female. **a** AP radiograph of the left knee shows an aggressive appearing osteolytic lesion (arrow) arising from the lateral aspect of the distal femoral metaphysis, with destruction of the lateral cortex. **b** Coronal short tau inversion recovery (STIR) MR image demonstrates a hyperintense lesion (arrow) in the distal femur metaphysis which extends across the growth plate into the epiphysis, and which disrupts the lateral cortex. **c** Coronal post-contrast fat-suppressed T1-weighted image shows that the lesion enhances with contrast (arrow). **d** Axial post-contrast fat suppressed T1-weighted image demonstrates inhomogeneous enhancement of the lesion, (arrow). **e** Axial DW-MRI reveals a hyperintense lesion (arrow) in the left distal femur. **f** Corresponding ADC map shows a hypointense signal (arrow), indicating restricted diffusion, with ADCmean of 1.25 × 10^−3^ mm^2^/s; ADCmin of 0.93 × 10^−3^ mm^2^/s, and ADCmax of 1.83 × 10^−3^ mm^2^/s

Chondroblastic osteosarcomas (Fig. 5) presented low-to-intermediate signal intensity on T1WI, high signal intensity on T2WI, and heterogeneous enhancement on T1C+ (Gd). On ADC maps, two lesions demonstrated lower signal intensity, and seven lesions demonstrated higher signal intensity than muscle. MRI characteristics and ADC map findings of different bone tumor types are provided (Table S1).

The median values of the ADCmean, ADCmin, and ADCmax for benign bone tumors [1.34 (1.13–1.83), 0.98 (0.73–1.34), and 1.80 (1.57–2.46) × 10^−3^ mm^2^/s, respectively] were significantly higher compared to malignant bone tumors [0.93 (0.78–1.03), 0.59 (0.43–0.72), and 1.35 (1.22–1.66) × 10^−3^ mm^2^/s, respectively; all *p* < 0.05] (Table 2). Although statistically significant differences in ADCmean, ADCmin, and ADCmax were observed between benign and malignant tumors, there was no interquartile range overlap for ADCmean (benign: 1.13–1.83 × 10^−3^ mm^2^/s; malignant: 0.78–1.03 × 10^−3^ mm^2^/s) or ADCmin (benign: 0.73–1.34 × 10^−3^ mm^2^/s; malignant: 0.43–0.72 × 10^−3^ mm^2^/s). In contrast, ADCmax demonstrated partial overlap between benign (1.57–2.46 × 10^−3^ mm^2^/s) and malignant tumors (1.22–1.66 × 10^−3^ mm^2^/s). This pattern suggests that ADCmean and ADCmin provide better separation between benign and malignant tumors in this cohort, whereas ADCmax is less discriminatory.

**Table 2:** ADC values for different types of benign and malignant bone tumors included in our study. Values are presented as median (interquartile range)

Type of bone tumor	ADCmean (× 10^−3^mm^2^/s)	ADCmin (× 10^−3^mm^2^/s)	ADCmax (× 10^−3^mm^2^/s)
**Benign (*N* = 48)**	**1.34 (1.13—1.83)**	**0.98 (0.73—1.34)**	**1.80 (1.57—2.46)**
Fibrous dysplasia (*N* = 28)	1.20 (1.10—1.30)	0.82 (0.69—0.92)	1.64 (1.45—1.94)
Enchondroma (*N* = 9)	2.21 (1.90—2.26)	1.55 (1.38—1.71)	2.57 (2.19—2.80)
Non ossifying fibroma (*N* = 11)	1.55 (1.45—1.99)	1.28 (1.15—1.42)	1.78 (1.70—2.48)
**Malignant (*N* = 48)**	**0.93 (0.78—1.03)**	**0.59 (0.43—0.72)**	**1.35 (1.22—1.66)**
Ewing sarcoma (*N* = 17)	0.73 (0.67—0.95)	0.35 (0.25—0.51)	1.31 (1.21—1.44)
Chondroblastic osteosarcoma (*N* = 9)	1.23 (1.19—1.26)	0.78 (0.68—0.95)	1.75 (1.46—1.82)
Osteoblastic osteosarcoma (*N* = 22)	0.88 (0.82—1.02)	0.61 (0.55—0.68)	1.29 (1.21- 1.46)

The average ADCmean of all benign lesions was 1.49 ± 0.43 × 10^−3^ mm^2^/s. In descriptive analysis, fibrous dysplasia exhibited lower ADC values among benign bone tumors, enchondroma demonstrated higher values, and non-ossifying fibromas showed intermediate values.

The average ADCmean of all malignant lesions was 0.93 ± 0.21 × 10^−3^ mm^2^/s. Among malignant bone tumors, Ewing sarcoma exhibited comparatively lower ADC values, chondroblastic osteosarcoma demonstrated higher values, and osteoblastic osteosarcomas showed intermediate values. These trends are reported descriptively and were not the result of formal statistical testing. (Table 3).

**Table 3:** ADC values for different types of benign and malignant bone tumors included in our study. Values are presented as mean ± standard deviation (SD)

Type of bone tumor	ADCmean (×10^−3^mm^2^/s)	ADCmin (×10^−3^mm^2^/s)	ADCmax (×10^−3^mm^2^/s)
**Benign (*N* = 48)**	**1.49 ± 0.43**	**1.05 ± 0.39**	**1.95 ± 0.50**
Fibrous dysplasia (*N* = 28)	1.24 ± 0.23	0.82 ± 0.23	1.74 ± 0.41
Enchondroma (*N* = 9)	2.10 ± 0.23	1.51 ± 0.29	2.48 ± 0.37
Non ossifying fibroma (*N* = 11)	1.65 ± 0.36	1.25 ± 0.36	2.04 ± 0.50
**Malignant (*N* = 48)**	**0.93 ± 0.21**	**0.58 ± 0.24**	**1.44 ± 0.32**
Ewing sarcoma (*N* = 17)	0.79 ± 0.19	0.38 ± 0.19	1.37 ± 0.33
Chondroblastic osteosarcoma (*N* = 9)	1.21 ± 0.12	0.82 ± 0.25	1.74 ± 0.31
Osteoblastic osteosarcoma (*N* = 22)	0.92 ± 0.12	0.62 ± 0.14	1.38 ± 0.24

Benign bone tumors exhibited significantly higher ADC values than malignant bone tumors (Table 3). All three ADC parameters (ADCmean, ADCmin, and ADCmax) were significantly higher in benign tumors compared to malignant tumors (*p* < 0.05 for all).

**Fig. 6:**
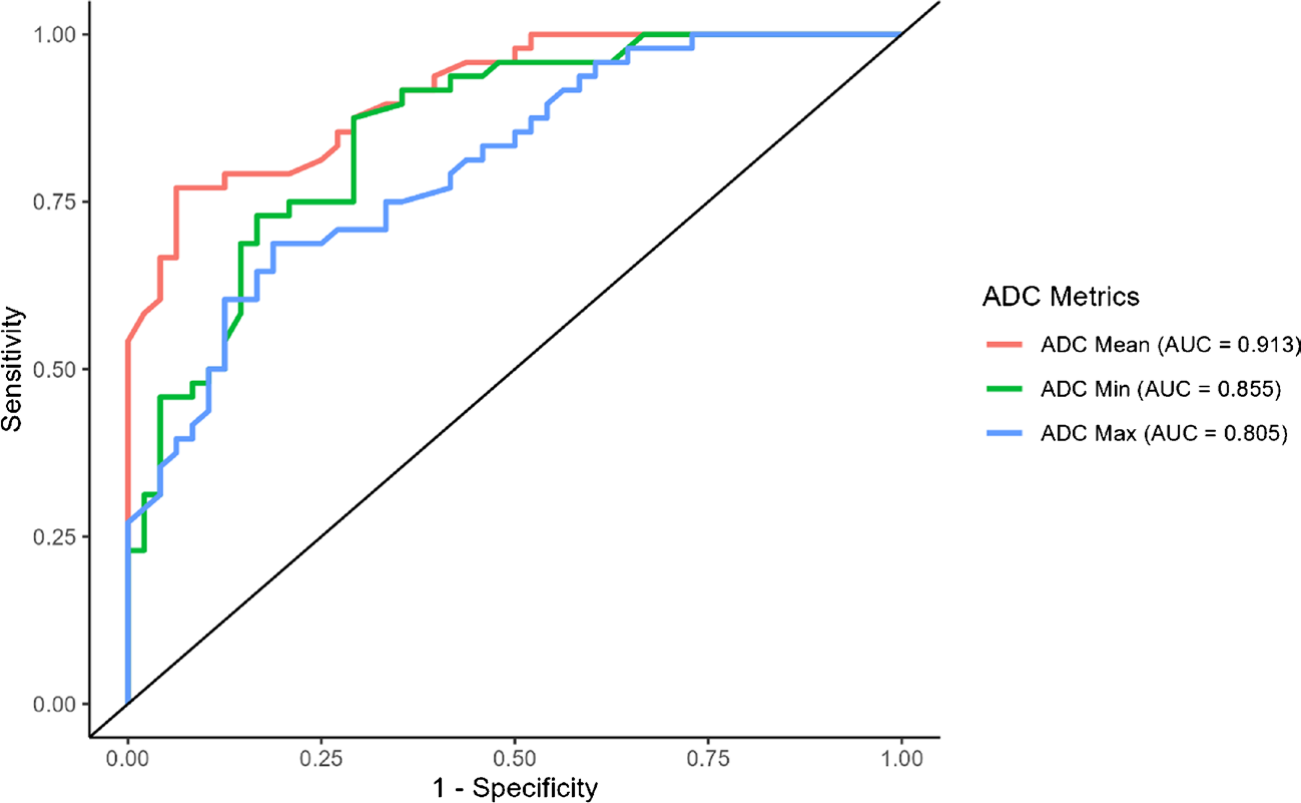
Receiver operating characteristic (ROC) curves demonstrating the accuracy of different ADC metrics (ADCmean, ADCmin, and ADCmax) in distinguishing benign vs malignant bone tumors. The x-axis (1—specificity) represents the false-positive rate, while the y-axis (sensitivity) represents the true-positive rate. ADCmean (red curve) has the highest area under the curve (AUC = 0.913), indicating the highest diagnostic accuracy among the three metrics; ADCmin (green curve) follows closely (AUC = 0.855), whereas ADCmax (blue curve) has the lowest accuracy (AUC = 0.805)

Receiver operating characteristic (ROC) curves were generated to assess the diagnostic accuracy (Fig. 6). An ADCmean cutoff of 1.04 (0.94–1.15) × 10^−3^ mm^2^/s yielded a sensitivity of 77.08% (95% CI: 62.80%–87.0%), a specificity of 93.75% (95% CI: 81.90%–98.0%), and an AUC of 0.91 (95% CI: 0.86–0.97) for differentiating benign and malignant tumors. An ADCmin cutoff of 0.82 (0.65–0.98) × 10^−3^ mm^2^/s yielded a sensitivity of 87.5% (95% CI: 74.40%–94.40%), a specificity of 70.83% (95% CI: 56.20%–82.10%), and an AUC of 0.86 (95% CI: 0.78–0.93). ADCmax cutoff of 1.48 (1.18–1.78) × 10^−3^ mm^2^/s yielded a sensitivity of 68.75% (95% CI: 54.0%–80.5%), a specificity of 81.25% (95% CI: 67.30%–90.10%), and an AUC of 0.81 (95% CI: 0.72–0.89) (Table 4). Alternative ADC cutoff values for differentiating benign from malignant bone lesions are provided (Table S2).

**Table 4:** Optimal ADC cutoff values, corresponding sensitivity, specificity, AUC, and *p*-values for differentiating benign and malignant primary bone tumors

Parameter	Optimal cutoff (×10^−3^mm^2^/s) [95% CI]	AUC [95% CI]	Sensitivity % [95% CI]	Specificity % [95% CI]	*p*-value
ADCmean	1.04 (0.94–1.15)	0.91 (0.86–0.97)	77.08 (62.80–87.0)	93.75 (81.90–98.0)	<0.05
ADCmin	0.82 (0.65–0.98)	0.86 (0.78–0.93)	87.50 (74.40–94.40)	70.83 (56.20–82.10)	<0.05
ADCmax	1.48 (1.18–1.78)	0.81 (0.72–0.89)	68.75 (54.00–80.50)	81.25 (67.30–90.10)	<0.05

Optimal cutoff = defined as the point at which the Youden index, the sum of sensitivity and specificity, was maximized. All estimates are reported with 95% confidence intervals (CI). AUC comparisons: Mean vs Min, *p* = 0.058; Mean vs Max, *p* = 0.38

## Discussion

Our study showed that ADC values can be used to differentiate between benign and malignant primary bone tumors in pediatric patients. To the best of our knowledge, this study is the first to compare ADC values of benign and malignant primary bone tumors in children. Previous research combined data from adult and pediatric patients [12, 14, 15], focused solely on ADCmean [10, 16] or examined both primary and metastatic bone tumors [11, 12, 15].

Various authors have described ADC values for distinct categories of bone tumors: Rao et al. reported that Ewing sarcoma had the lowest ADCmean (0.7 × 10^−3^ mm^2^/s) among various malignant bone tumors, including Ewing’s sarcoma, osteosarcoma, giant cell tumor, chondrosarcoma, malignant fibrous histiocytosis, plasmacytoma, and telangiectatic osteosarcoma [10]. This may be due to the histological composition of Ewing sarcoma, which includes small round cells with minimal intervening stroma. The tightly packed nature of these cells creates a microenvironment that significantly limits water diffusion, leading to very low ADC values. By contrast, chondroblastic malignancies had high ADCmean values. Rao et al. reported an ADCmean of 2.1 × 10^−3^ mm^2^/s for chondrosarcomas [10] and Setiawati et al. reported an ADCmean of 1.47 × 10^−3^ mm^2^/s for chondroblastic osteosarcoma [17]. Setiawati et al. reported that ADCmean of chondroblastic osteosarcoma were significantly higher compared to that of osteoblastic osteosarcoma (0.99 × 10^−3^ mm^2^/s), fibroblastic osteosarcoma (1.0 × 10^−3^ mm^2^/s) and telangiectasis osteosarcoma (0.92 × 10^−3^ mm^2^/s) [17]. This can be explained by the dispersion of tumor cells within the chondroid matrix, allowing water molecules to move more freely, reducing diffusion restriction and resulting in higher ADC values [18].

Reports in the literature described higher ADCmean values of benign cystic and vascular lesions. Nouh et al. reported that unicameral bone cysts had an ADCmean of 2.69 ± 0.52 × 10^−3^ mm^2^/s and that hemangiomas had an ADCmean of 1.67 ± 0.18 × 10^−3^ mm^2^/s [16]. Rao et al. reported that aneurysmal bone cysts had an ADCmean of 2.0 ± 0.1 × 10^−3^ mm^2^/s [10]. Usually, the diagnosis of these lesions is not challenging due to their characteristic appearance on conventional MRI sequences.

Pekcevik et al. reported ADCmean values of 1.98 × 10^−3^ mm^2^/s for enchondromas, ADCmean values of 1.23 × 10^−3^ mm^2^/s for non-ossifying fibromas and ADCmean values of 1.41 × 10^−3^ mm^2^/s for fibrous dysplasia [19] The lower ADC values observed in fibrous dysplasia and non-ossifying fibromas are likely due to their fibrous tissue content, which restricts diffusion [19].

In accordance with our results, several investigators reported higher ADCmean values in benign bone tumors compared to malignant tumors in patients with a wide age range. Ahlawat et al. reported higher ADCmean values of benign tumors (1.68 ± 0.4 × 10^−3^ mm^2^/s) compared to our study [12], likely because their analyses included four bone cysts and a hemangioma among the 18 benign tumors analyzed. They also reported higher ADCmean values of malignant tumors (1.13 ± 0.36 × 10^−3^ mm^2^/s) compared to our study [12]. This might have been due to inclusion of an angiosarcoma and lack of Ewing sarcomas in their patient population.

Wang et al. reported significantly higher ADCmean values of benign lesions (fibrous tumors, Langerhans cell histiocytosis and giant cell tumor) compared to malignant bone tumors (Ewing sarcoma, osteosarcoma, chondrosarcoma, plasmacytoma, lymphoma, chordoma and metastases) in both adult and pediatric patients, with an ADCmean cutoff value of 1.1 × 10^−3^ mm^2^/s [15]. Kotb et al. reported that benign lesions (osteoid osteoma, chondroblastoma, osteochondroma, fibrous tumors, enchondroma and hemangioma) had ADCmean values 1.27–2.19 × 10^−3^ mm^2^/s (mean-1.75 × 10^−3^ mm^2^/s), while malignant bone tumors (osteosarcoma, chondrosarcoma, myeloma, lymphoma, Ewing’s sarcoma, plasmacytoma, leukemia, ameloblastoma, and metastases) had ADCmean values 0.9–2.05 × 10^−3^ mm^2^/s (mean-1.39 × 10^−3^ mm^2^/s) [14].

All of these previous studies investigated tumors in patients with a wide age range of 4–80 years [12], 2–73 years [16], 1–92 years [15], and 4–65 years [14]. In contrast, our study concentrated exclusively on solid bone tumors in pediatric patients with an age of 18 years or less. This is important because the underlying hematopoietic marrow exhibits intrinsic restricted diffusion and different tumor types are prevalent in the pediatric population.

Neubauer et al. reported an ADCmean threshold of 1.03 × 10^−3^ mm^2^/s for differentiating 34 benign from 10 malignant bone lesions in children [11]. Our investigation in a larger cohort of 96 children found an almost identical ADCmean threshold of 1.04 (0.94–1.15) × 10^−3^ mm^2^/s. Additionally, we expanded these analyses to investigate the sensitivity and specificity of ADCmin and ADCmax.

Our study had several limitations. Firstly, it is a single-center study, which may impact the generalizability of the results. However, this approach ensured consistent, high-quality MRI data, which minimizes the chance that observed differences were influenced by technical factors. Although our study population is limited to 96 cases, this investigation represents the largest cohort study to date examining ADC values of primary bone tumors in pediatric patients. The equal distribution between benign (*n* = 48) and malignant (*n* = 48) cases reduced imbalance bias of our analyses and improved the rigor of our statistical comparisons. Conventional MRI remains foundational for the characterization of bone tumors, exploiting differences in T1- and T2-weighted signal properties to characterize internal architecture and tissue composition. However, substantial signal overlap among certain benign and malignant bone tumors limits the diagnostic specificity. Contrast-enhanced MRI further aids in delineating tumor margins, differentiating solid tumors from cystic lesions, and identifying areas of necrosis. However, its use may be contraindicated in patients with contrast allergy or impaired renal function. Quantitative ADC measurements derived from diffusion-weighted imaging provide an objective, reproducible surrogate of tissue microstructure and may mitigate these limitations, particularly in diagnostically equivocal cases. The integration of ADC into routine bone tumor MRI protocols requires further validation through studies comparing its sensitivity and specificity with those of conventional and contrast-enhanced MRI.

In summary, this study demonstrates that ADC can differentiate benign and malignant primary bone tumors in pediatric patients, with an ADCmean cutoff of 1.04 (0.94–1.15) × 10^−3^ mm^2^/s having a higher diagnostic accuracy with a sensitivity of 77.08% (62.8–87.0%), specificity of 93.75% (81.9–98.0%), and an AUC of 0.91 (0.86–0.97). Utilizing ADC measurements may improve the accuracy of bone tumor diagnosis and support informed clinical decision-making.

## Supplementary Information

Below is the link to the electronic supplementary material.

Supplementary file1 (DOCX 457 KB)

## Data Availability

The imaging data were obtained at Lucile Packard Children’s Hospital and Stanford Healthcare and are not publicly available to protect patient privacy. The de-identified data can be obtained from the authors upon reasonable request and with approval from the institutional review board.
