# Supplementary material for: Apparent diffusion coefficient can assist in differentiating between benign and malignant primary bone tumors in pediatric patients
Source: Skeletal Radiol. 2025 Oct 29;55(3):621–31. doi: 10.1007/s00256-025-05060-8 (PMC12847184; doi:10.1007/s00256-025-05060-8)
Supplement: Supplementary file 1 — Supplementary file1 (DOCX 457 KB) [file 256_2025_5060_MOESM1_ESM.docx]

#
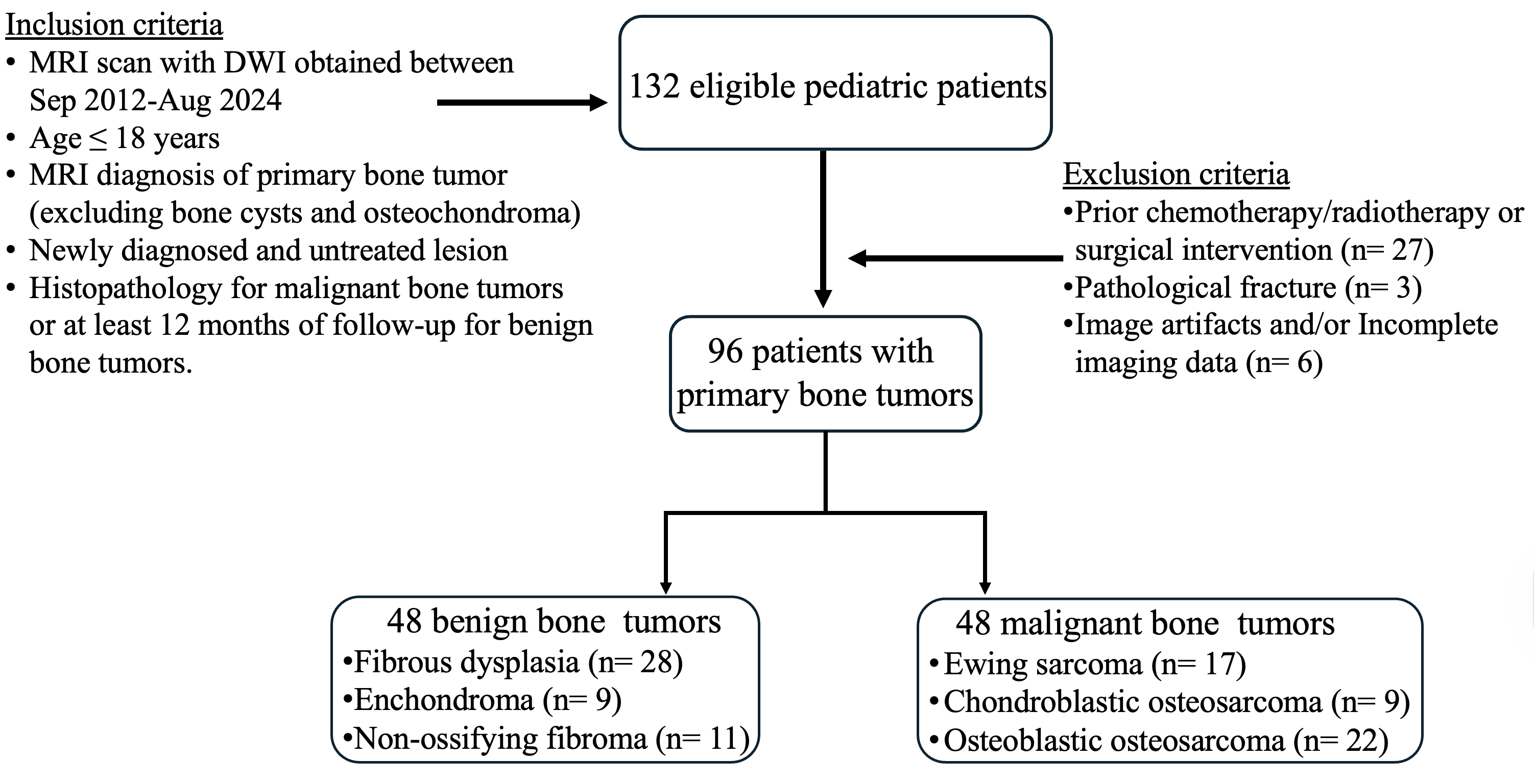


**Fig. S1** Flow diagram illustrating the patient selection process for the study.

# Table S1. MRI characteristics and ADC map findings of different bone tumor types

| Tumor Type | T1-weighted Imaging (T1WI) | T2-weighted Imaging (T2WI) | Contrast-enhanced T1-weighted Imaging (T1C+ [Gd]) | ADC Map Findings |
| --- | --- | --- | --- | --- |
| Fibrous dysplasia | Moderately low signal intensity | Intermediate signal intensity | Heterogeneous moderate enhancement | 7 lesions showed lower signal intensity and 21 lesions showed higher signal intensity than muscle |
| Non-ossifying fibroma | Low signal intensity | Intermediate-to-heterogeneously high signal intensity often with a low-signal peripheral rim | Heterogeneous enhancement | 1 lesion showed lower signal intensity and 10 lesions showed higher signal intensity than muscle |
| Enchondroma | Intermediate-to-low signal intensity | Heterogeneously high signal intensity | Moderate heterogeneous enhancement | All lesions showed higher signal intensity than muscle |
| Ewing sarcoma | Low-to-intermediate signal intensity | Heterogeneously high signal intensity | Moderate-to-high heterogeneous enhancement | All lesions showed lower signal intensity than muscle |
| Osteoblastic osteosarcoma | Inhomogeneous hypointense (non-mineralized: intermediate; mineralized: low) | Inhomogeneous hyperintense (non-mineralized: high; mineralized: low) | Heterogeneous enhancement | All lesions showed lower signal intensity than muscle |
| Chondroblastic osteosarcoma | Low to intermediate signal intensity | High signal intensity | Heterogeneous enhancement | 2 lesions showed lower signal intensity; 7 lesions showed higher signal intensity than muscle |

#

# Table S2. Optimal and alternative ADC cut-off values for differentiating benign and malignant bone lesions.

| Group | ADC Cut-off | AUC (95% CI) | Sensitivity %  (95% CI) | Specificity %  (95% CI) |
| --- | --- | --- | --- | --- |
| Mean | 1.15 | 0.78  (0.69-0.86) | 83.3%  (69.6-91.6) | 72.9%  (58.3-83.8) |
|  | 0.94 | 0.75  (0.68-0.82) | 50.0%  (35.9-64.1) | 100%  (100-100) |
|  | **Optimal = 1.04** | **0.91  (0.86-0.97)** | **77.08%  (62.80-87.0)** | **93.75%  (81.90-98.0)** |
| Min | 0.65 | 0.76  (0.68-0.85) | 66.6%  (51.9-78.7) | 85.4%  (72.0-93.0) |
|  | 0.98 | 0.73  (0.65-0.81) | 95.80%  (84.3-99.0) | 50.0%  (35.9-64.1) |
|  | **Optimal = 0.82** | **0.86  (0.78-0.93)** | **87.50%  (74.40-94.40)** | **70.83%  (56.20-82.10)** |
| Max | 1.18 | 0.55  (0.51-0.60) | 10.4%  (4.3-23.1) | 100%  (100-100) |
|  | 1.78 | 0.67  (0.58-0.76) | 83.3%  (69.6-91.6) | 50.0%  (35.9-64.1) |
|  | **Optimal = 1.48** | **0.81  (0.72-0.89)** | **68.75%  (54.0-80.50)** | **81.25%  (67.3-90.1)** |

Footnote: The optimal cutoff of ADC values to differentiate benign and malignant bone tumors was defined as the point at which the Youden index, the sum of sensitivity and specificity, was maximized.
